# PTSD symptomics: network analyses in the field of psychotraumatology

**DOI:** 10.1080/20008198.2017.1398003

**Published:** 2017-12-08

**Authors:** Cherie Armour, Eiko I. Fried, Miranda Olff

**Affiliations:** ^a^ Psychology Research Institute, Ulster University, Coleraine, Northern Ireland; ^b^ Department of Psychology, University of Amsterdam, Amsterdam, The Netherlands; ^c^ Department of Psychiatry, Academic Medical Center, University of Amsterdam, Amsterdam, The Netherlands; ^d^ Department of Psychiatry, Arq Psychotrauma Expert Group, Diemen, The Netherlands

**Keywords:** PTSD, network analysis, symptomics, symptoms, psychopathology

## Abstract

Recent years have seen increasing attention on posttraumatic stress disorder (PTSD) research. While research has largely focused on the dichotomy between patients diagnosed with mental disorders and healthy controls — in other words, investigations at the level of diagnoses — recent work has focused on psychopathology symptoms. Symptomics research in the area of PTSD has been scarce so far, although several studies have focused on investigating the network structures of PTSD symptoms. The present special issue of EJPT adds to the literature by curating additional PTSD network studies, each looking at a different aspect of PTSD. We hope that this special issue encourages researchers to conceptualize and model PTSD data from a network perspective, which arguably has the potential to inform and improve the efficacy of therapeutic interventions.

## La síntomática del TEPT: Análisis de red en el campo de la psicotraumatología

Los últimos años han visto una atención creciente en la investigación sobre el trastorno por estrés postraumático (TEPT). Si bien la investigación se ha centrado principalmente en la dicotomía entre pacientes diagnosticados con trastornos mentales y controles sanos –en otras palabras, investigaciones a nivel de diagnóstico–, el trabajo reciente se ha centrado en los síntomas psicopatológicos. La investigación sintomática en el área del TEPT ha sido escasa hasta el momento, aunque varios estudios se han centrado en investigar las estructuras de la red de los síntomas del TEPT. Este número especial de la EJPT se agrega a la literatura con estudios adicionales de la red de TEPT, cada uno analizando un aspecto diferente del TEPT Esperamos que este número especial anime a los investigadores a conceptualizar y modelar los datos del TEPT desde una perspectiva de red, lo que sin duda tiene el potencial de informar y mejorar la eficacia de las intervenciones terapéuticas.

## PTSD症候群： 心理创伤领域的网络分析

近年来对创伤后应激障碍研究的关注有所提高。大量研究关注诊断病人和健康控制组的区分上——换句话说，研究在诊断层面——近期工作集中在心理病理症状上。在PTSD领域的症候研究目前还很缺乏，尽管一些研究已经开始关注PTSD症状的网络结构。这本EJPT的特刊收录额外的PTSD网络研究，各自关注PTSD的不同方面。我们希望这次特刊可以鼓励研究者从网络视角看待和建立PTSD数据模型，虽然还存在争议，但这有可能为提供治疗干预效果提供信息。

## 1. Introduction

Recent years have seen increasing attention on posttraumatic stress disorder (PTSD) research, with a focus on the diagnostic criteria for PTSD, (early) intervention research, PTSD biomarkers, and many others (e.g. Armour, Műllerová, & Elhai, 2015; Colvonen et al., 2017; Kassam-Adams, 2014; Kehle-Forbes & Kimerling, 2017; Molnar et al., 2017; Olff, Van Zuiden, & Bakker, 2015; Wisco et al., 2016). This has led to important discoveries and has inspired some work on improving prevention and intervention strategies for trauma-affected individuals (Brief et al., 2013; Hilton et al., 2017; Horn, Charney, & Feder, 2016; Markowitz et al., 2015; Olff, Armour et al., 2015). At the same time, many important questions remain unresolved or under-researched, such as aetiological and symptomatological differences of patients, how to best predict future adverse outcomes, and whether particular PTSD symptoms play important roles and might be viable intervention targets.

While research has largely focused on the dichotomy between patients diagnosed with mental disorders and healthy controls – in other words, investigations at the level of diagnoses – recent work has focused on psychopathology symptoms. One reason for this is that disorder-level data are often highly heterogeneous: patients suffering from mental disorders such as PTSD or depression have many different symptom presentations (Fried & Nesse, 2015a; Glück, Knefel, Tran, & Lueger-Schuster, 2016; Olbert, Gala, & Tupler, 2014; Young, Lareau, & Pierre, 2014). Heterogeneity has also led to ongoing discussions about reliability and validity of DSM and ICD diagnoses (Insel, 2013). Information on the patient’s particular story, and a focus on the specific symptoms the patient exhibits, may provide crucial information. While the importance of specific symptoms has long been acknowledged in clinical practice, where clinicians often base their treatment decisions on symptoms and not diagnoses (Kim & Ahn, 2002; Waszczuk et al., 2017), clinical sciences lag somewhat behind.

Symptom-based analyses are on the rise for many disorders such as major depression (see review; Fried & Nesse, 2015b), bipolar disorder (Pfennig et al., 2015), and psychosis (Bentall, Wickham, Shevlin, & Varese, 2012). A new research framework entitled ‘Symptomics’ was proposed recently that aims to describe such studies under one framework, and complement diagnosis-level research with more detailed research on symptoms (Fried, 2017). Symptomics has three cornerstones:

1. The relationship of *individual* symptoms with important variables such as risk factors, biomarkers, impairment of functioning, and treatment response (Bentall et al., 2012; Costello, 1993; Fried & Nesse, 2015b; Hieronymus, Emilsson, Nilsson, & Eriksson, 2016; Persons, 1986);
2. The analysis of the potentially causal relations among symptoms in symptom networks (Borsboom, 2017; Cramer, Waldorp, van der Maas, & Borsboom, 2010; Wichers, Wigman, & Myin-Germeys, 2015);
3. Gaining better understanding of psychopathology by investigating personalized processes at the level of individuals instead of heterogeneous groups of patients (Fisher & Boswell, 2016; Molenaar, 2004).

Symptomics research in the area of PTSD has been scarce so far, although several studies have focused on investigating the network structures of PTSD symptoms (Afzali et al., 2017; Armour, Fried, Deserno, Tsai, & Pietrzak, 2017; Bryant et al., 2017; Fried et al., 2017; McNally et al., 2015; Mitchell et al., 2017). The present special issue of *EJPT* adds to the literature by curating four additional PTSD network studies, each looking at a different aspect of PTSD.

## 2. In this issue

In ‘Symptoms of posttraumatic stress disorder in a clinical sample of refugees: a network analysis’, Spiller and colleagues (Spiller et al., 2017) explored the network structure and centrality of DSM-5 PTSD symptoms in a sample of 151 severely traumatized adult refugees attending two outpatient clinics for victims of torture and war in Switzerland. As such, this was the first application of state-of-the-art network methodology to a refugee population. The authors’ primary focus was to identify the strongest connections among symptoms as well as the most and least central symptoms in the network. The authors found that some connections were substantially stronger than others (defined as being stronger than at least half of all associations in the networks): hypervigilance with exaggerated startle response, intrusions with difficulties falling asleep, and irritability/outbursts of anger with reckless/self-destructive behaviour. The most central symptom was emotional cue reactivity; the least central symptom trauma-related amnesia. These findings in a cross-sectional and highly clinical refugee population highlight the potential importance of re-experiencing symptoms, particularly trauma-related emotional cue reactivity. However, the authors pointed out that their results should be understood as hypothesis generation and interpreted with caution due to the limited power and therefore accuracy of parameter estimates that result from a fairly small sample size.

In the paper ‘Making connections: exploring the centrality of posttraumatic stress symptoms and covariates after a terrorist attack’, Skogbrott Birkeland and Heir (2017) identified the most central symptoms of DSM-IV PTSD and their interconnections in a sample of 190 ministerial employees 10 months after the 2011 Oslo bombings. Interestingly, the authors also included covariates such as sex, neuroticism, and social support in the network. About 25% of the participants met the criteria for a probable PTSD diagnosis. In the estimated network, the strongest association emerged between the three symptom pairs of intrusive thoughts and nightmares, hypervigilance and feeling easily startled, and feeling detached and feeling emotionally numb. The symptom of feeling emotionally numb was identified as the most central symptom in the network, although the authors also state that the order of centrality estimates must be interpreted with some care due to limited power. Similar to prior studies (McNally et al., 2015) and also results of papers published in this special issue (Spiller et al., 2017), trauma-related amnesia was identified as the least central symptom. When adding covariates to the network, a strong negative connection emerged between social support and sleep problems, and female sex was strongly related to physiological cue reactivity, highlighting the importance of including non-symptom nodes in network models, and justifying the authors’ attempt to determine why and how covariates could be related PTSD symptomatology. Birkeland and Heir thereby address one of the most crucial challenges of the emerging field of network sciences in psychopathology research: modelling variables that go beyond symptoms (Fried & Cramer, 2017).

Glück, Knefel, and Lueger-Schuster (2017) in their paper ‘A network analysis of anger, shame, proposed ICD-11 PTSD, and different types of childhood trauma in foster care settings in a sample of adult survivors’ utilized network analysis to examine the relationships between childhood abuse, anger, shame, and the proposed ICD-11 PTSD symptoms in a sample of 220 adult survivors of institutional abuse. Many participants met criteria for comorbid mental disorders, over half of the sample met ICD-11 PTSD criteria, and 16.9% met the criteria for complex PTSD. To circumvent the problems associated with small sample sizes, the authors first conducted the analysis using scale-level data to identify those constructs that are most important in the relationship of trauma, PTSD, anger, and shame. The results highlight anger rumination, trait anger, emotional abuse, and PTSD re-experiencing as central nodes. Interestingly, trait anger was not connected with any type of childhood abuse, nor was it connected with other lifetime traumatic events, suggesting that it may be a result of some external events. Another possibility is that the connection was not very strong, and was not discovered due to the fairly small sample size. Anger rumination was identified as a ‘promoter of the network’, due to its high closeness centrality, and the authors suggested it as a potential treatment target. The most central scales, along with the two remaining ICD-11 PTSD subscales avoidance and sense of threat, were subsequently utilized in item-level network analysis. The most central node in this latter analysis was an anger rumination item – ‘getting “worked up” thinking about upsetting things in past’ – and was suggested as the bridge in the relationships between anger and ICD-11 PTSD. In other words, it may be the item responsible for the co-occurrence of PTSD and anger. This item, along with two other anger rumination items (both of which were related to meaningful past events and life in general) were most closely connected with the emotional abuse and the PTSD symptoms. The authors also examined the modularity of their network using community structure analysis and discovered clearly separable subgroups of constructs, largely supporting the validity of the included scales. The study concluded that treatments aimed at alleviating the burden of posttraumatic symptomatology may be effective if transdiagnostic phenomena, such as anger rumination, are addressed.

Finally, in a sample of 179 US adult survivors of childhood sexual abuse, McNally, Heeren, and Robinaugh (2017) in ‘A Bayesian network analysis of posttraumatic stress disorder symptoms in adults reporting childhood sexual abuse’ extend the existing PTSD network analysis research by applying two distinct network models. Firstly, the authors computed an undirected regularized partial correlation network (based on the DSM-IV PTSD symptoms). These network models estimate conditional dependence relations among items: what is the association between A and B after partialling out the influence of C? In such undirected networks, suggest McNally et al. (2017), the causal relationships between symptoms cannot be inferred. Thus, in the second step, the authors utilized a Bayesian approach to computing a directed acyclic network, which leads to a directed network structure often referred to as a ‘causal skeleton’. In the undirected network, none of the symptoms could be considered to be significantly more central than others due to the low stability and robustness statistics resulting from the small sample size. The directed network structure highlighted the potentially important role of physiological arousal in response to reminders of the traumatic experiences. McNally et al. suggest that targeting this symptom in interventions could potentially lead to an improvement in other PTSD symptoms, at least in adult survivors of childhood sexual abuse. For a broader discussion on the application of Bayesian methods to the field of PTSD research, see *Bayesian statistics in the field of psychotraumatology* (Van de Schoot, Schalken, & Olff, 2017).

## 3. Conclusions

The above papers represent a significant contribution to the literature on posttraumatic stress, as viewed from a symptom-level perspective. We hope that this special issue encourages researchers to conceptualize and model PTSD data from a network perspective, which arguably has the potential to inform and improve the efficacy of therapeutic interventions (Borsboom, 2017; Cramer et al., 2010; Hayes & Strauss, 1998; McNally, 2016). The methodological field of psychological network psychometrics has moved remarkably quickly from visualizing correlation matrices in 2010 (Cramer et al., 2010) to using sophisticated statistical models in 2014 (Van Borkulo et al., 2014). The gap between clinical sciences and methodology is slowly closing, in part due to several tutorial papers (Costantini et al., 2017; Epskamp, Borsboom, & Fried, 2017; Epskamp & Fried, 2017) that have enabled clinical researchers to apply network models to a large number of disorders (for a review see Fried, van Borkulo et al., 2017).

However, several crucial challenges still lie ahead, some of which are described in detail elsewhere (Fried & Cramer, 2017). First, as illustrated in this special issue, PTSD network studies in subclinical or clinical samples are often limited to small samples, leading to uncertainty surrounding parameter estimates (such as the connections among items in the network, or the centrality estimates), precluding reliable generalizations. Studies with larger samples, on the other hand, often feature general population samples that may not be too informative about the processes in patients. A potential way forward is the analysis of large clinical datasets (e.g. Fried, Eidhof et al., 2017). Second, it is crucial that researchers start focusing on the assessment and analysis of dynamic, temporal data (Bringmann et al., 2016 ; Epskamp et al., 2017; Hamaker & Wichers, 2017). This allows the field to move from modelling cross-sectional group-level data to modelling the temporal dynamics of causal systems across time, and might bring us closer to developing novel recommendations for intervention or prevention strategies (Bos et al., 2017). Third, more attention to modelling the dynamics of causal systems also allows a renewed focus on personalized medicine, seeing that time-series network models are not limited to modelling the symptom dynamics of groups of patients, but can also be used to obtain idiographic network structures for individual patients (Epskamp, van Borkulo et al., 2017; Fisher & Boswell, 2016; Kroeze et al., 2017). Fourth, there is evidence that biological markers are differentially related to specific psychopathology symptoms (e.g. inflammatory markers and depression symptoms; Jokela, Virtanen, & Batty, 2016). Including (neuro-) biological measures associated with PTSD and its comorbid disorders in network analyses might move the field beyond ‘symptomics’ and help us better understand the complex relationships between neurobiological alterations (over time) and the development of trauma related psychopathology (Olff & van Zuiden, 2017). This is consistent with recent calls to include variables beyond symptoms in psychopathology network models (Fried & Cramer, 2017; Jones, Heeran, & McNally, 2017).

We believe that the network approach to psychopathology has been embraced so quickly by the community because it reflects how patients and clinicians think about many mental disorders: as dynamic systems of causal influences and vicious circles. Interestingly, this lies in contrast with the frameworks adopted by the DSM and ICD that understand symptoms as passive consequences of underlying disorders. We are looking forward to seeing whether the analysis of individual symptoms and causal dynamics can lead to significant changes in how mental disorders are measured, modelled, categorized, and diagnosed.
